# Supplementary material for: Multi‐dataset identification of innovative feature genes and molecular mechanisms in keratoconus
Source: J Cell Mol Med. 2024 Sep 19;28(18):e70079. doi: 10.1111/jcmm.70079 (PMC11412914; doi:10.1111/jcmm.70079)
Supplement: Supplementary file 2 — Material S2. [file JCMM-28-e70079-s002.zip › 7.GSEA analysis/3.DBNDD1/KEGG_APOPTOSIS.html]

Details for gene set KEGG\_APOPTOSIS[GSEA]

|  || Dataset | DBNDD1.group.cls#h\_versus\_l |
| Phenotype | group.cls#h\_versus\_l |
| Upregulated in class | h |
| GeneSet | KEGG\_APOPTOSIS |
| Enrichment Score (ES) | 0.1554153 |
| Normalized Enrichment Score (NES) | 0.62184644 |
| Nominal p-value | 0.9656566 |
| FDR q-value | 1.0 |
| FWER p-Value | 1.0 |
Table: GSEA Results Summary

  

Fig 1: Enrichment plot: KEGG\_APOPTOSIS      
 Profile of the Running ES Score & Positions of GeneSet Members on the Rank Ordered List

  

| SYMBOL | TITLE | RANK IN GENE LIST | RANK METRIC SCORE | RUNNING ES | CORE ENRICHMENT || 1 | PIK3R5 | na | 35 | 0.708 | 0.0862 | Yes |
| 2 | PPP3R2 | na | 1210 | 0.361 | 0.0968 | Yes |
| 3 | IL3RA | na | 1219 | 0.360 | 0.1409 | Yes |
| 4 | IRAK3 | na | 2771 | 0.261 | 0.1283 | Yes |
| 5 | PRKAR1B | na | 2920 | 0.255 | 0.1554 | Yes |
| 6 | IL3 | na | 3952 | 0.215 | 0.1521 | No |
| 7 | TNFRSF10C | na | 6465 | 0.138 | 0.0966 | No |
| 8 | PIK3R1 | na | 6912 | 0.126 | 0.0993 | No |
| 9 | APAF1 | na | 8227 | 0.099 | 0.0736 | No |
| 10 | CHP2 | na | 8667 | 0.091 | 0.0722 | No |
| 11 | FASLG | na | 8688 | 0.091 | 0.0828 | No |
| 12 | RIPK1 | na | 8963 | 0.086 | 0.0856 | No |
| 13 | AKT2 | na | 9388 | 0.080 | 0.0832 | No |
| 14 | CASP8 | na | 9432 | 0.079 | 0.0917 | No |
| 15 | CASP10 | na | 9729 | 0.075 | 0.0924 | No |
| 16 | NTRK1 | na | 10107 | 0.070 | 0.0902 | No |
| 17 | IRAK1 | na | 10588 | 0.063 | 0.0841 | No |
| 18 | CASP6 | na | 10699 | 0.062 | 0.0886 | No |
| 19 | ENDOG | na | 10775 | 0.061 | 0.0939 | No |
| 20 | FADD | na | 10784 | 0.061 | 0.1012 | No |
| 21 | BID | na | 11063 | 0.057 | 0.1002 | No |
| 22 | ATM | na | 11600 | 0.051 | 0.0910 | No |
| 23 | PIK3R2 | na | 11699 | 0.050 | 0.0944 | No |
| 24 | DFFB | na | 11787 | 0.049 | 0.0979 | No |
| 25 | XIAP | na | 12176 | 0.045 | 0.0923 | No |
| 26 | CASP3 | na | 13339 | 0.035 | 0.0632 | No |
| 27 | BAX | na | 13486 | 0.034 | 0.0632 | No |
| 28 | MYD88 | na | 13863 | 0.031 | 0.0561 | No |
| 29 | TP53 | na | 13962 | 0.030 | 0.0570 | No |
| 30 | PIK3R3 | na | 14144 | 0.029 | 0.0554 | No |
| 31 | CAPN1 | na | 14897 | 0.024 | 0.0366 | No |
| 32 | CFLAR | na | 15483 | 0.019 | 0.0221 | No |
| 33 | PRKX | na | 15799 | 0.017 | 0.0151 | No |
| 34 | CASP7 | na | 15997 | 0.016 | 0.0114 | No |
| 35 | TRADD | na | 16696 | 0.011 | -0.0074 | No |
| 36 | PIK3CA | na | 17073 | 0.009 | -0.0171 | No |
| 37 | PIK3CB | na | 18338 | 0.000 | -0.0536 | No |
| 38 | PPP3R1 | na | 18349 | 0.000 | -0.0538 | No |
| 39 | PRKAR2A | na | 18901 | -0.004 | -0.0693 | No |
| 40 | CHUK | na | 18953 | -0.004 | -0.0702 | No |
| 41 | AKT1 | na | 19066 | -0.005 | -0.0729 | No |
| 42 | PRKAR1A | na | 19604 | -0.010 | -0.0872 | No |
| 43 | BIRC2 | na | 19822 | -0.011 | -0.0921 | No |
| 44 | CASP9 | na | 19881 | -0.012 | -0.0923 | No |
| 45 | CYCS | na | 20083 | -0.014 | -0.0964 | No |
| 46 | CAPN2 | na | 20301 | -0.016 | -0.1007 | No |
| 47 | BAD | na | 20453 | -0.017 | -0.1030 | No |
| 48 | TNFRSF10A | na | 20481 | -0.017 | -0.1017 | No |
| 49 | PRKACA | na | 20814 | -0.020 | -0.1087 | No |
| 50 | IRAK2 | na | 21779 | -0.030 | -0.1329 | No |
| 51 | TRAF2 | na | 22056 | -0.033 | -0.1368 | No |
| 52 | IKBKB | na | 22300 | -0.035 | -0.1395 | No |
| 53 | CHP1 | na | 22407 | -0.036 | -0.1381 | No |
| 54 | PPP3CA | na | 22457 | -0.037 | -0.1350 | No |
| 55 | TNFRSF10B | na | 22768 | -0.040 | -0.1390 | No |
| 56 | TNFRSF1A | na | 22847 | -0.041 | -0.1361 | No |
| 57 | IRAK4 | na | 22853 | -0.041 | -0.1312 | No |
| 58 | PPP3CB | na | 22975 | -0.043 | -0.1294 | No |
| 59 | IKBKG | na | 23063 | -0.044 | -0.1265 | No |
| 60 | EXOG | na | 23536 | -0.050 | -0.1340 | No |
| 61 | AIFM1 | na | 23712 | -0.052 | -0.1326 | No |
| 62 | DFFA | na | 24375 | -0.062 | -0.1441 | No |
| 63 | TNFSF10 | na | 24501 | -0.063 | -0.1399 | No |
| 64 | BIRC3 | na | 24545 | -0.064 | -0.1333 | No |
| 65 | FAS | na | 24662 | -0.066 | -0.1285 | No |
| 66 | PIK3CD | na | 24971 | -0.070 | -0.1288 | No |
| 67 | ENDOD1 | na | 25839 | -0.084 | -0.1434 | No |
| 68 | NFKB1 | na | 25842 | -0.084 | -0.1330 | No |
| 69 | PRKAR2B | na | 26168 | -0.091 | -0.1312 | No |
| 70 | TNFRSF10D | na | 26206 | -0.092 | -0.1210 | No |
| 71 | NGF | na | 26508 | -0.098 | -0.1176 | No |
| 72 | BCL2L1 | na | 26569 | -0.099 | -0.1071 | No |
| 73 | PRKACB | na | 26730 | -0.102 | -0.0992 | No |
| 74 | PPP3CC | na | 26734 | -0.102 | -0.0867 | No |
| 75 | CSF2RB | na | 26951 | -0.107 | -0.0798 | No |
| 76 | TNF | na | 27164 | -0.111 | -0.0721 | No |
| 77 | PIK3CG | na | 27455 | -0.118 | -0.0660 | No |
| 78 | NFKBIA | na | 27480 | -0.118 | -0.0521 | No |
| 79 | PRKACG | na | 28125 | -0.134 | -0.0542 | No |
| 80 | RELA | na | 28616 | -0.147 | -0.0503 | No |
| 81 | IL1B | na | 28762 | -0.150 | -0.0359 | No |
| 82 | IL1A | na | 31036 | -0.222 | -0.0742 | No |
| 83 | IL1RAP | na | 31082 | -0.223 | -0.0480 | No |
| 84 | BCL2 | na | 31549 | -0.242 | -0.0316 | No |
| 85 | MAP3K14 | na | 32153 | -0.273 | -0.0154 | No |
| 86 | IL1R1 | na | 33185 | -0.343 | -0.0029 | No |
| 87 | AKT3 | na | 33656 | -0.385 | 0.0309 | No |
Table: GSEA details [plain text format]

  

Fig 2: KEGG\_APOPTOSIS      
 Blue-Pink O' Gram in the Space of the Analyzed GeneSet

  

Fig 3: KEGG\_APOPTOSIS: Random ES distribution      
 Gene set null distribution of ES for **KEGG\_APOPTOSIS**
